# When telepressure never sleeps: psychological/spatial detachment as mediators to sleep quality in digital nomads

**DOI:** 10.3389/fpubh.2026.1788479

**Published:** 2026-05-11

**Authors:** Zixuan Xu, Qi Cao, Ziran Zhou, Yimo Han, Chao Yang, Hui-Ling Hu

**Affiliations:** 1 School of Business, Yangzhou University, Yangzhou, China; 2 Graduate School of International Studies, Department of East Asian Studies, Hanyang University, Seoul, Republic of Korea; 3 Department of Mathematics, Faculty of Science, National University of Singapore, Singapore, Singapore; 4 School of Creative Arts, Beijing University of Financial Technology, Beijing, China; 5 Institute of Creative Design and Management, National Taipei University of Business, Taoyuan, Taiwan, China

**Keywords:** digital nomads, psychological detachment, psychological resilience, sleep quality, spatial detachment, telepressure

## Abstract

**Introduction:**

Grounded in the Stressor–Detachment Model and the Job Demands–Resources Theory, this study examines how telepressure—the perceived obligation to stay constantly connected—affects the sleep quality of digital nomads through psychological and spatial detachment, while testing the moderating role of psychological resilience. We hypothesized that telepressure reduces psychological and spatial detachment, which in turn impairs sleep quality, and that psychological resilience buffers these effects.

**Methods:**

Using cross-sectional survey data from 539 digital nomads in China, this study employed partial least squares structural equation modeling (PLS-SEM) to test the hypothesized direct, mediating, and moderating relationships. Telepressure, psychological detachment, spatial detachment, and psychological resilience were assessed using established multi-item scales, and sleep quality was measured using the Pittsburgh Sleep Quality Index (PSQI). Job demands were included as a control variable.

**Results:**

Telepressure exerted significant negative effects on both psychological detachment (β = −0.265, *p* < 0.001) and spatial detachment (β = −0.153, *p* = 0.003), whereas its direct effect on sleep quality was not significant (β = −0.028, *p* = 0.567). Mediation analyses revealed that telepressure indirectly impaired sleep quality through both psychological detachment (indirect effect = −0.039, 95% CI [−0.073, −0.009]) and spatial detachment (indirect effect = −0.019, 95% CI [−0.042, −0.002]). Psychological resilience positively moderated the relationship between spatial detachment and sleep quality (β = 0.159, *p* = 0.006) but did not significantly moderate the psychological detachment–sleep quality link.

**Discussion:**

The findings highlight the pivotal role of dual detachment mechanisms in the recovery process and illuminate the health risks of telepressure in flexible, technology-mediated work contexts. Individuals with higher resilience appear better able to maintain behavioral and environmental boundaries for effective recovery. The results emphasize the necessity of supporting both psychological and spatial detachment to sustain workplace health and sleep quality among digital nomads.

## 1 Introduction

In the post-COVID-19 pandemic era, the normalization of remote and “work-from-anywhere” arrangements has led an increasing number of knowledge workers to adopt a digital nomad lifestyle—working across locations while maintaining online collaboration. Digital nomads are commonly defined as individuals who leverage digital technologies to perform location-independent work while maintaining high levels of mobility (1, 2). From a work design perspective, such arrangements are characterized by high autonomy and blurred boundaries between work and non-work domains. Meanwhile, the pervasive integration of information and communication technologies (ICTs) and digital platforms has intensified the “always-on” availability norm and telepressure, defined as the felt obligation and compulsion to respond to messages immediately (3, 4). This phenomenon is particularly pronounced in cross-time-zone collaborations, where availability demands and irregular working hours reinforce one another, undermining individuals’ ability to achieve psychological and spatial detachment during non-work hours, thereby impairing sleep and recovery (5).

Recent research further indicates that digital nomads’ high mobility and weakened local embeddedness make it more challenging for them to sustain structured routines and clear work–life boundaries (6, 7). As Cook (8) points out, while digital nomadism offers substantial flexibility, it simultaneously demands heightened self-discipline and boundary management. Accordingly, this study focuses on the telepressure–detachment–sleep mechanism, situating the analysis within the digital nomad context to examine its implications for health and recovery.

Sleep quality serves as a fundamental cornerstone of workplace health and performance. Systematic reviews and longitudinal studies have demonstrated that remote work arrangements continuously alter sleep behaviors—such as delayed bedtimes and wake times, and increased time spent in bed—with these effects showing a growing and persistent trend (9). Improving sleep quality, in turn, has been shown to enhance psychological well-being (10). Within the workplace context, sleep quality is closely linked to outcomes such as stress, exhaustion, and daytime dysfunction (11). Therefore, clarifying how telepressure influences sleep quality through detachment experiences holds direct practical significance for Workplace Health and Wellbeing (12, 13).

Existing research has consistently demonstrated that telepressure is associated with poorer recovery experiences, reduced sleep quality, and greater exhaustion (3, 4). Furthermore, both theoretical and empirical studies have identified psychological detachment as a key mechanism in the stress–health process (14). However, three major research gaps remain to be addressed. First, most studies have focused on general remote employees, with limited empirical evidence specifically examining digital nomads. Furthermore, although telepressure and recovery processes have been widely examined in Western and general remote work contexts, empirical evidence from the Chinese context remains limited. Given China’s highly digitalized work environment, strong norms of responsiveness, and widespread use of mobile communication platforms (e.g., WeChat) for work-related coordination, employees may experience more pervasive and intensified telepressure compared to other contexts. These contextual characteristics suggest that the mechanisms linking telepressure, detachment, and sleep quality may operate differently or more strongly in China. Therefore, examining this model in the Chinese context is both theoretically and practically meaningful, as it extends existing theories to a high-intensity digital communication environment and provides context-specific insights into recovery and well-being among digital workers. Second, prior research has predominantly emphasized psychological detachment, while giving insufficient attention to the comparative effects of psychological and spatial (physical/behavioral) detachment (15). Third, evidence regarding whether personal resources—such as psychological resilience—can buffer the adverse effects of telepressure on detachment and sleep remains fragmented and often confined to specific occupational groups. These gaps constrain our understanding of which boundary mechanisms most effectively protect sleep and who is more resilient under high availability pressure.

Grounded in the Stressor–Detachment Model and the Job Demands–Resources (JD–R) Theory, this study posits that telepressure, as an information-related demand and availability norm, acts as a stressor that undermines two boundary mechanisms—psychological detachment and spatial detachment—thereby impairing sleep quality (5, 16). At the same time, psychological resilience, conceptualized as a personal resource, may buffer the negative effects of telepressure either in the “stressor → detachment” or “detachment → sleep” pathways. Accordingly, this study proposes a dual-mediation model, in which telepressure affects sleep quality through both psychological and spatial detachment, and further examines the moderating and conditional indirect effects of psychological resilience. In addition, following Boundary Theory, this study operationalizes spatial detachment through concrete spatial and behavioral boundary strategies—such as maintaining a designated workspace or engaging in shutdown rituals—to clearly differentiate it from psychological detachment (15). Accordingly, this study assumes a causal mechanism in which telepressure undermines detachment, thereby impairing sleep quality.

## 2 Literature review

### 2.1 Theoretical foundation

This study is grounded in four key theoretical frameworks—the Job Demands–Resources (JD–R) Theory, the Stressor–Detachment Model, Boundary Theory, and the Conservation of Resources (COR) Theory—to construct the mechanism linking telepressure, detachment, and sleep quality. According to the JD–R theory, telepressure represents a form of digital work demand that depletes personal energy and harms well-being, while personal resources such as psychological resilience function as buffers against these adverse effects (16). The Stressor–Detachment Model and Recovery Theory further emphasize that psychological detachment and relaxation facilitate the restoration of depleted resources, and that sleep quality serves as a critical recovery outcome (5). Boundary Theory extends this perspective by introducing the spatial dimension of recovery, highlighting that maintaining physical and behavioral boundaries helps individuals separate work and non-work roles (15, 17). Meanwhile, the COR Theory posits that psychological resilience enables individuals to maintain functioning and recovery under high-demand conditions (18).

In summary, this study proposes that telepressure indirectly affects sleep quality by undermining both psychological and spatial detachment, while psychological resilience moderates and buffers these effects. Together, these mechanisms form an integrated “Demand–Detachment–Sleep” framework, illustrating how digital work stressors influence recovery and well-being.

### 2.2 The negative effect of telepressure on psychological detachment

According to the Job Demands–Resources (JD–R) Theory and the Stressor–Detachment Model, the norm of constant availability and the compulsion for instant response—collectively referred to as telepressure—constitute a form of digital work demand. Its core mechanism lies in the persistent cognitive and emotional occupation of work cues during non-work hours, which hinders individuals’ ability to psychologically disengage from work contexts (5, 16). From the perspective of Boundary Theory, telepressure fosters frequent “micro-boundary crossings” and a perceived obligation to remain responsive, making it difficult for individuals to maintain clear distinctions between work and non-work domains (17, 19). Such blurred boundaries weaken employees’ capacity for evening psychological detachment and recovery. Moreover, continuous expectations of availability trigger cognitive rumination and physiological arousal, diverting attentional resources toward potential work-related stimuli. This heightened vigilance reduces individuals’ ability to deliberately refrain from work-related thoughts during leisure time, thereby creating unfavorable conditions for subsequent sleep and recovery (5, 20).

Empirical findings and meta-analytic evidence support the mechanisms described above. Telepressure has been shown to be significantly associated with lower recovery experiences and psychological detachment, poorer sleep quality, and unfavorable work–life outcomes (3, 4). Diary and survey studies further reveal that after-hours email or smartphone use, combined with norms of immediate responsiveness, diminishes evening psychological detachment while increasing fatigue and tension. Notably, these effects stem not only from the duration of technology use but also from the perceived expectation of availability itself (14, 21).

Based on the theoretical and empirical evidence discussed above, the following hypothesis is proposed:

*H1*: Telepressure has a negative effect on psychological detachment.

### 2.3 The negative effect of telepressure on sleep quality

Theoretically, telepressure represents a form of digital work demand that extends work-related cues and a sense of obligation into non-work hours, creating persistent cognitive occupation and emotional arousal. According to the health impairment process of the Job Demands–Resources (JD–R) theory, prolonged exposure to such demands depletes individual resources and undermines health. Within the frameworks of the Stressor–Detachment Model and Recovery Theory, this sustained activation first weakens psychological detachment, subsequently disrupting the deactivation process before sleep and the continuity of nighttime rest. These disruptions manifest as prolonged sleep latency, more frequent awakenings, and a decline in subjective sleep quality (5, 20, 22). From a boundary perspective, when expectations of responsiveness and availability intrude into evening routines, they interrupt bedtime rituals and environmental cues that normally facilitate relaxation. This intrusion heightens sympathetic arousal and ruminative thinking before sleep, thereby deteriorating overall sleep quality.

Empirical evidence aligns with the mechanisms described above. Telepressure has been found to be significantly associated with poorer sleep quality, partly mediated by insufficient recovery experiences, such as lower levels of psychological detachment (3, 4). Moreover, studies have shown that evening communication and smartphone use related to availability norms reduce psychological detachment and predict poorer sleep, greater fatigue, and increased exhaustion the following day (14, 21, 23).

Based on the theoretical and empirical evidence discussed above, the following hypothesis is proposed:

*H2*: Telepressure has a negative effect on sleep quality.

### 2.4 The negative effect of telepressure on spatial detachment

From the perspective of Boundary Theory (17, 19), individuals maintain distinct work and non-work roles through spatial, temporal, and psychological boundaries. Among these, spatial detachment refers to the creation of an uninterrupted environment through tangible physical and behavioral boundaries—such as maintaining a designated workspace, turning off work devices, or engaging in offline rituals (15). However, telepressure, driven by the obligation to respond instantly and the norm of constant availability, makes it difficult for individuals to sustain stable spatial separation and behavioral transitions. When work-related messages can reach employees anywhere and at any time, digital nomads are particularly prone to managing work tasks in cafés, bedrooms, or while in transit. This blurs the distinction between workspaces and living spaces, eroding the physical and behavioral boundaries necessary for effective recovery (24).

According to the stress–detachment model (5), such persistent availability pressure disrupts the recovery process at both physical and behavioral levels. Even when individuals leave the workplace or shut down their computers, they may remain in a “quasi-work state” due to anticipated message notifications or anxiety about not responding. Under these conditions, the mechanism of spatial detachment fails, preventing non-work environments from fulfilling their restorative function (22). At the same time, Job Demands–Resources (JD–R) theory suggests that excessive digital work demands erode individuals’ resources, making it more difficult for them to invest the necessary energy in maintaining boundaries through effective boundary management (16).

Empirically, Barber et al. (3) and Derks et al. (21) both found that the frequency of work-related mobile device use and expectations for prompt responses reduce employees’ behavioral segmentation at home. Matthews et al. (15) also confirmed that when spatial and behavioral boundary strategies are insufficient, work intrusion and fatigue increase significantly. For digital nomads, telepressure not only undermines psychological detachment but also disrupts the sense of “being offline” that is maintained through spatial arrangements and daily rituals, thereby weakening recovery quality and sleep conditions. Integrating theory and empirical evidence, the following hypothesis is proposed:

*H3*: Telepressure has a negative effect on spatial detachment.

### 2.5 The effects of psychological detachment and spatial detachment on sleep quality

Within the frameworks of the Stressor–Detachment Model and Recovery Theory, psychological detachment and spatial detachment are regarded as two core mechanisms that promote recovery and enhance sleep quality (20, 25). Psychological detachment refers to an individual’s ability to temporarily stop thinking about or dealing with work-related matters during non-work time, thereby achieving mental disengagement from work. Spatial detachment, in contrast, emphasizes the creation of physical and behavioral boundaries—such as having a designated workspace, turning off devices, or engaging in offline rituals—to facilitate role transitions and environmental separation (17, 19).

Psychological detachment can reduce physiological arousal and cognitive rumination, allowing the mind and body to enter a state of rest and restoration (22). Conversely, when detachment is insufficient, work-related worries and a persistent sense of responsibility continue to occupy attentional resources, leading to prolonged sleep onset latency, increased nighttime awakenings, and reduced sleep continuity (14). This phenomenon is particularly pronounced in remote and mobile work contexts, where blurred temporal and spatial boundaries make individuals more susceptible to remaining in a continual “mental on-duty mode” (3).

On the other hand, spatial detachment provides a concrete behavioral recovery strategy. According to Boundary Theory, individuals can reduce the spillover of work cues by separating work and non-work environments, thereby allowing personal spaces to regain their restorative and relaxation functions (15, 24). Recovery Theory further suggests that when the external environment clearly differentiates between work and rest domains, situational arousal is lowered, which facilitates sleep-related recovery (5). In contrast, when work extends into the bedroom or other private spaces, the restorative cues of these environments are weakened, leading to heightened pre-sleep arousal and difficulty falling asleep (22).

A growing body of empirical research has confirmed the importance of these dual mechanisms. Sonnentag and Fritz (20) found that individuals with higher levels of psychological detachment report significantly better subjective sleep quality and greater next-day vitality. Wendsche and Lohmann-Haislah (14) meta-analysis likewise indicated that psychological detachment is a significant predictor of sleep quality and well-being. Derks et al. (21) further demonstrated that using smartphones at home in the evening to handle work-related tasks substantially reduces sleep quality that night. In addition, Kossek et al. (24) showed that individuals who maintain clear spatial boundaries experience lower fatigue and higher levels of recovery. For highly mobile digital nomads—whose work-life boundaries are inherently blurred—maintaining both psychological and spatial detachment can partially offset the negative sleep effects generated by telepressure.

Taken together, psychological detachment can be viewed as an internal cognitive–emotional relaxation mechanism, whereas spatial detachment represents an external environmental–behavioral recovery condition. Together, these two mechanisms form a dual protective system that enhances sleep quality, indicating that individuals who effectively disengage from work after hours, and who reinforce this disengagement through clear spatial boundaries and “after-work rituals,” can meaningfully improve their sleep quality and overall health.

*H4*: Psychological detachment positively influences sleep quality.

*H5*: Spatial detachment positively influences sleep quality.

### 2.6 The mediating roles of psychological detachment and spatial detachment

According to the Stressor–Detachment Model, job demands influence recovery and health outcomes by undermining individuals’ ability to detach from work during non-work hours (5). Within this framework, telepressure—as a key form of digitalized job demand—creates a persistent expectation of “constant availability” and “immediate responsiveness,” thereby generating prolonged attentional occupation and heightened psychological arousal (4). This sustained activation of work-related thoughts makes it difficult for individuals to cease cognitive processing related to work after hours, resulting in impaired psychological detachment and subsequently disrupting the sleep recovery process (3, 22).

At the same time, from the perspective of Boundary Theory (17, 19), telepressure affects not only the psychological process of “deactivation” but also disrupts spatial and behavioral boundaries. Because digital communication tools allow work messages to reach individuals at any moment, people are often compelled to respond even in non-work settings (e.g., bedrooms, restaurants, cafés), rendering spatial detachment strategies—such as turning off devices or engaging in offline rituals—ineffective (15, 24). When spatial boundaries become blurred and work cues spill into personal environments, the recovery process is hindered, ultimately manifesting as poorer sleep quality and heightened next-day fatigue (21).

Empirical studies have provided preliminary support for this dual mediation mechanism. The integrated findings of Barber et al. (3) and Wendsche and Lohmann-Haislah (14) indicate that insufficient psychological detachment significantly explains the indirect effects of telepressure on sleep and exhaustion. Likewise, Matthews et al. (15) and Kossek et al. (24) emphasize that deficits in spatial and behavioral boundary strategies exacerbate work interference and health impairment. Together, these findings suggest that psychological and spatial detachment jointly constitute a dual mediating pathway linking job demands to health outcomes.

Based on the theoretical and empirical evidence, the following hypotheses are proposed:

*H6*: Psychological detachment mediates the relationship between telepressure and sleep quality.

*H7*: Spatial detachment mediates the relationship between telepressure and sleep quality.

### 2.7 The moderating role of psychological resilience

According to Conservation of Resources (COR) Theory, individuals rely on their psychological and social resources to maintain functioning and prevent depletion when encountering high demands or stressors (18). Psychological resilience, as a cultivable personal resource, encompasses elements such as emotion regulation, problem-focused coping, attentional shifting, and self-efficacy, enabling individuals to regain equilibrium rapidly in stressful environments (26). Similarly, the Job Demands–Resources (JD–R) framework posits that personal resources can buffer the adverse effects of job demands on health outcomes (16).

In the high-arousal and high-demand context created by telepressure, psychological resilience is expected to play a moderating role in the relationship between detachment and sleep. Individuals with high resilience can more effectively regulate emotional responses and restore attentional focus through adaptive strategies; thus, even when detachment is limited, they are still able to mitigate the disruptive effects of arousal and anxiety on the sleep onset process, maintaining better sleep quality. In contrast, individuals with low resilience are more likely to experience excessive rumination and emotional exhaustion when detachment is insufficient, resulting in more pronounced impairment in sleep quality (5, 18).

Moreover, psychological resilience may also influence the effectiveness of spatial detachment. Highly resilient individuals tend to establish clearer role boundaries and greater self-discipline; even under the availability pressure induced by telepressure, they are more capable of adhering to offline behaviors and pre-sleep rituals, thereby sustaining the stability of restorative environments. Conversely, individuals with low resilience may be more likely to break boundaries due to perceived expectations or anxiety, causing spatial detachment strategies to fail (15, 24).

Integrating COR and JD–R theories, this study posits that psychological resilience serves as a buffering mechanism between detachment and sleep, amplifying the protective effects of detachment on sleep quality. Accordingly, the following hypotheses are proposed:

*H8*: Psychological resilience moderates the relationship between psychological detachment and sleep quality, such that the positive effect of psychological detachment is stronger among individuals with higher resilience.

*H9*: Psychological resilience moderates the relationship between spatial detachment and sleep quality, such that the positive effect of spatial detachment is stronger among individuals with higher resilience.

### 2.8 Job demands as a control variable

In this study, Job Demands are included as a control variable to account for the general influence of work-related stress on individuals’ psychological and physiological states. According to the Job Demands–Resources (JD–R) Theory (27, 28), job demands refer to the sustained effort and psychological costs required to perform work tasks, including workload, time pressure, and emotional demands. High levels of job demands can lead to energy depletion, restricted recovery, and deteriorated health outcomes (29).

Prior research has shown a significant negative relationship between job demands and sleep quality. High workloads and long working hours increase physiological arousal and cognitive rumination, thereby prolonging sleep onset and reducing sleep efficiency (22). Job demands also affect individuals’ ability to detach from work: when task pressure persists, employees find it difficult to stop work-related thinking even during non-work time (5).

Therefore, when examining the relationships among telepressure, psychological/spatial detachment, and sleep quality, controlling for job demands helps eliminate the confounding influence of general work strain. This ensures that the observed effects primarily reflect the additional burden generated by digital stressors (telepressure) rather than the impact of traditional job stress. Such a design enhances the internal validity of the model and clarifies the unique health-related mechanisms through which telepressure operates in contemporary remote and mobile work environments.

**Figure 1:**
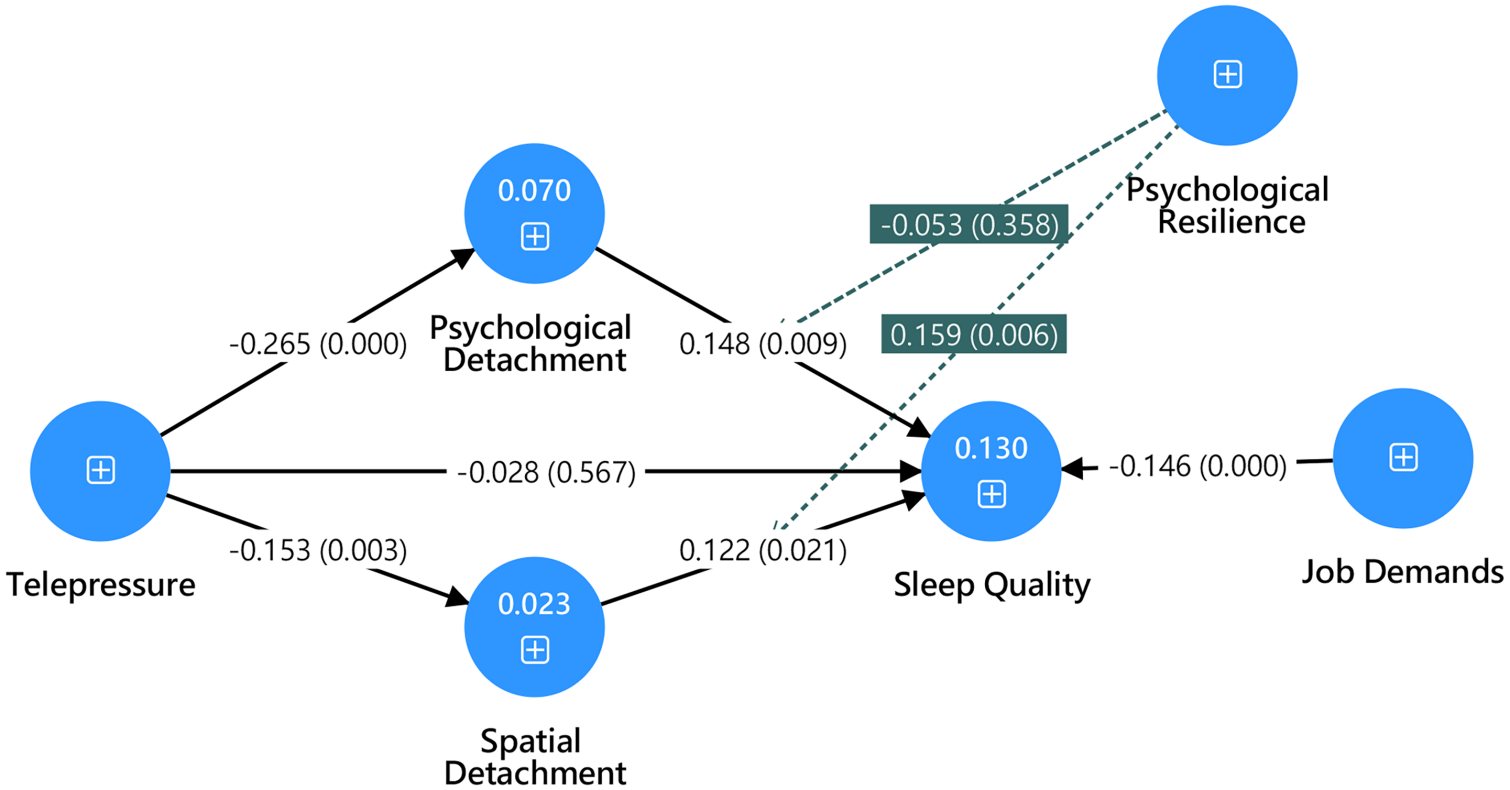
Research model.

The research model proposed in this study is presented in Figure 1.

## 3 Research method

### 3.1 Research participants and data collection

Based on the assumption that telepressure reduces psychological and spatial detachment, which in turn impairs sleep quality, and that psychological resilience buffers these effects, this study targeted digital nomads in China, this study targeted digital nomads in China and employed online convenience sampling with a one-time cross-sectional survey. Inclusion criteria required participants to: (1) be at least 18 years old; (2) have engaged in remote work or location-independent living across at least two cities within the past 12 months; and (3) have weekly opportunities for remote work. Individuals engaging only in short-term tourism or non-work travel were excluded. Data were collected from 2025/09/01 to 2025/09/30 through recruitment postings in coworking spaces, digital nomad communities/forums, and via social media sharing. Following the recommendations of Hair, Risher, Sarstedt, and Ringle (30) for PLS-SEM, sample size determination should be based on statistical power rather than the simple “10-times rule.” With *α* = 0.05, power = 0.80, and approximately 4–5 predictors for each endogenous construct—assuming a small to medium effect size (f^2^ ≈ 0.10–0.15)—a minimum sample of roughly 150–200 participants is required. Considering the complexity of this study, which includes mediation and moderation effects, as well as planned data quality screening, the target number of valid responses was set to approximately 400, ensuring robust estimation and reliable empirical results.

### 3.2 Measurement instruments

All constructs in this study were measured using self-report questionnaires and were divided into two sections: demographic background and research constructs. Demographic variables included gender, age, education level, occupation, weekly hours of remote work, and place of residence. Except for sleep quality, all research constructs were assessed using a 7-point Likert scale (1 = strongly disagree, 7 = strongly agree). All items were translated using a “translation–back translation” procedure and refined through a pilot test with 30–50 digital nomads to ensure semantic clarity and appropriate item sequencing.

Telepressure (TP) was measured using the 6-item Telepressure Scale developed by Barber and Santuzzi (4) (e.g., “When I receive messages from others, I find it difficult to focus on other things”). This construct reflects perceived obligations of constant availability and immediate responsiveness to work-related communication. Psychological Detachment (PD) was assessed using the four items from the “psychological detachment” dimension of the Recovery Experience Questionnaire (20) (e.g., “During non-work time, I do not think about work at all”), capturing the degree of cognitive and emotional disengagement during off-job hours. Spatial Detachment (SD) was measured using five adapted items from the physical/behavioral boundary tactics of Matthews et al. (15) (e.g., “I designate specific physical spaces for work and non-work,” “I put away or turn off work devices during non-work time”), assessing offline boundaries established through physical separation and ritualized behaviors.

Sleep Quality (SQ). Sleep quality was assessed using the Chinese version of the Pittsburgh Sleep Quality Index (PSQI) (31). The PSQI is a widely validated instrument that evaluates sleep quality over the past month across seven components: subjective sleep quality, sleep latency, sleep duration, habitual sleep efficiency, sleep disturbances, use of sleep medication, and daytime dysfunction. Following the standard PSQI scoring procedure, each component was scored from 0 to 3 and summed to produce a global score ranging from 0 to 21, with higher scores indicating poorer sleep quality. Consistent with common practice in behavioral and health research, the PSQI global score was used as the indicator representing overall sleep quality in the structural model. Prior studies have widely adopted the PSQI global index as a composite measure of sleep quality rather than modeling the individual components separately (31, 32). In addition, to ensure robustness, supplementary analyses were conducted using the commonly applied PSQI cutoff score (>5) to classify poor sleep quality. Psychological Resilience (PR) was assessed using the 10-item CD-RISC-10 (26, 33) (e.g., “I am able to bounce back quickly when facing stress”), serving as the moderating variable. Finally, Job Demands (JD) were measured with the five-item Quantitative Workload Inventory (QWI) (34) (e.g., “I often work under time pressure”), included as a control variable. All measurement instruments used in this study were adapted from established and previously validated scales. These instruments have demonstrated satisfactory reliability and validity in prior research. In addition, the measurement properties were further assessed in the present study using reliability and validity analyses.

### 3.3 Data analysis

This study employed PLS-SEM to validate the research model, with model estimation conducted using SmartPLS. Data preprocessing included sample exclusion (e.g., failure on screening questions, attention checks, or excessively short response times), handling of missing data, and identification of extreme values. The measurement model was evaluated based on the following criteria: indicator loadings ≥ 0.70, Cronbach’s alpha (*α*) and composite reliability (CR) ≥ 0.70, average variance extracted (AVE) ≥ 0.50, and HTMT < 0.85. Variance inflation factors (VIF < 5) were also examined to assess multicollinearity among constructs. For the structural model, 5,000 bootstrap resamples were used to estimate path coefficients and 95% confidence intervals, and R^2^ values were reported. Mediation analyses assessed the indirect effects of telepressure → psychological/spatial detachment → sleep quality, along with direct and total effects. Moderation analyses examined the interaction terms in which psychological resilience (PR) moderates the paths TP → PD and TP → SD, with simple slope plots generated at ±1 SD. Control variables included Job Demands (JD) and essential demographics (e.g., age, gender, weekly hours of remote work).

## 4 Research results

### 4.1 Analysis of participants’ background characteristics

A total of 539 valid responses were obtained. In terms of gender, females constituted the majority (70.7%), while males accounted for 29.3%. Regarding age distribution, the largest groups were participants aged 40–49 (30.1%) and 18–24 (26.2%), indicating representation across multiple age cohorts. With respect to educational attainment, most respondents held a bachelor’s degree (56.0%), followed by those with a graduate degree or above (16.9%) and junior college/associate degrees (15.4%), suggesting a relatively high level of education among participants. In terms of work patterns, most respondents reported engaging in 0–10 h of remote work per week (64.1%), indicating a predominantly hybrid work arrangement. At the same time, approximately one-third of the participants reported more than 20 h of remote work per week, reflecting a substantial subgroup with sustained remote work engagement. Overall, the sample demonstrates demographic diversity while maintaining characteristics broadly consistent with digital nomad populations reported in prior studies. Detailed distributions are presented in Table 1.

**Table 1:** Analysis of participants’ basic demographic information.

Category	Group	Frequency	Percentage
Gender	Male	158	29.3
	Female	381	70.7
Age	18–24	141	26.2
	25–29	66	12.2
	30–34	38	7.1
	35–39	50	9.3
	40–49	162	30.1
	50 and above	82	15.2
Educational level	Junior high	14	2.6
	High school	49	9.1
	Junior college	83	15.4
	University	302	56.0
	Graduate school	91	16.9
Weekly remote work hours	0–10 h	346	64.1
	11–19 h	52	9.6
	20–29 h	40	7.4
	30–39 h	35	6.5
	40–49 h	31	5.7
	50 h or more	35	6.5

### 4.2 Convergent validity

Following the recommendations of Fornell and Larcker (35) and Nunnally (36), the evaluation of the measurement model’s validity includes four indicators: factor loadings, composite reliability (CR), average variance extracted (AVE), and Cronbach’s *α*. The analysis results show that the factor loadings of all items range from 0.733 to 1.000, CR ranges from 0.897 to 0.963, and AVE ranges from 0.636 to 0.723 (AVE was not calculated for Sleep Quality as it is a single-item construct). Cronbach’s α values range from 0.862 to 0.960, all exceeding the recommended threshold of 0.7. Overall, the measurement model demonstrates good reliability and convergent validity, indicating that the scale effectively measures each construct with strong statistical robustness. See Table 2 for details.

**Table 2:** Convergent validity analysis.

Construct	Item	Factor loading	Cronbach’s alpha	Composite reliability (CR)	Average variance extracted (AVE)
Job demands	JD1	0.807	0.863	0.897	0.636
	JD2	0.872			
	JD3	0.754			
	JD4	0.733			
	JD5	0.815			
Psychological detachment	PD1	0.766	0.862	0.905	0.705
	PD2	0.831			
	PD3	0.890			
	PD4	0.866			
Psychological resilience	PR1	0.866	0.960	0.963	0.723
	PR2	0.866			
	PR3	0.886			
	PR4	0.843			
	PR5	0.830			
	PR6	0.868			
	PR7	0.843			
	PR8	0.863			
	PR9	0.819			
	PR10	0.817			
Spatial detachment	SD1	0.764	0.868	0.903	0.651
	SD2	0.822			
	SD3	0.880			
	SD4	0.752			
	SD5	0.808			
Telepressure	TE1	0.836	0.924	0.940	0.725
	TE2	0.872			
	TE3	0.876			
	TE4	0.864			
	TE5	0.792			
	TE6	0.865			
Sleep quality	SQ	1.000	—	—	—

### 4.3 Discriminant validity

In this study, discriminant validity for reflective constructs was assessed using the criterion proposed by Fornell and Larcker (35), which evaluates the square root of the average variance extracted (AVE). When the square root of each construct’s AVE is greater than its correlations with other constructs, adequate discriminant validity is established. The analysis results show that most constructs meet this criterion, indicating that each construct is distinct and capable of differentiating between concepts. Overall, the model demonstrates good discriminant validity. See Table 3 for details.

**Table 3:** Discriminant validity analysis.

Construct	Job demands	Psychological detachment	Psychological resilience	Sleep quality	Spatial detachment	Telepressure
Job Demands	**0.798**					
Psychological Detachment	−0.130	**0.839**				
Psychological Resilience	0.142	0.046	**0.850**			
Sleep Quality	−0.157	0.242	0.110	**1.000**		
Spatial Detachment	−0.015	0.507	0.125	0.228	**0.807**	
Telepressure	0.185	−0.266	−0.026	−0.110	−0.154	**0.851**

The bold values on the diagonal represent the square root of the average variance extracted (AVE) for each construct.

### 4.4 Common method bias

Harman’s single-factor test was conducted to examine the potential issue of common method bias. The exploratory factor analysis revealed that five factors with eigenvalues greater than 1 were extracted, explaining 70.934% of the total variance. The first factor accounted for 25.569% of the variance, which is below the suggested threshold of 50% (37). Therefore, common method bias was not considered a serious concern in this study, as shown in Table 4. However, it should be noted that Harman’s single-factor test has been criticized for its limited sensitivity in detecting common method variance. Thus, although the results suggest that common method bias is unlikely to substantially distort the findings, the results should be interpreted with caution. Future research is encouraged to adopt more rigorous procedures, such as marker-variable techniques, latent common method factor approaches, or time-lagged research designs, to further address potential common method bias.

**Table 4:** Common method bias.

Component	Initial Eigenvalues			Extraction sums of squared loadings			Rotation sums of squared loadings		
	Total	% of variance	Cumulative %	Total	% of variance	Cumulative %	Total	% of variance	Cumulative %
1	7.671	25.569	25.569	7.671	25.569	25.569	7.469	24.898	24.898
2	5.730	19.099	44.667	5.730	19.099	44.667	4.384	14.615	39.512
3	3.615	12.048	56.716	3.615	12.048	56.716	3.376	11.254	50.767
4	2.779	9.264	65.980	2.779	9.264	65.980	3.264	10.881	61.647
5	1.486	4.954	70.934	1.486	4.954	70.934	2.786	9.287	70.934

### 4.5 Hypothesis testing

The structural model was assessed using bootstrapping with 5,000 resamples to examine the proposed hypotheses. The results of direct effects hypothesis testing are presented in Table 5.

**Table 5:** Path relationship analysis.

Path relationship	Path coefficient	Standard deviation	*t*-value	*p-*value
Job Demands →Sleep Quality	−0.146	0.041	3.528	0.000
Psychological Detachment →Sleep Quality	0.148	0.057	2.610	0.009
Spatial Detachment→ Sleep Quality	0.122	0.053	2.304	0.021
Telepressure →Psychological Detachment	−0.265	0.045	5.836	0.000
Telepressure →Sleep Quality	−0.028	0.048	0.572	0.567
Telepressure → Spatial Detachment	−0.153	0.051	3.009	0.003

The path analysis results show that Job Demands have a significant negative effect on Sleep Quality (*β* = −0.146, t = 3.528, *p* < 0.001), indicating that higher job demands lead to poorer sleep quality. Both Psychological Detachment (*β* = 0.148, t = 2.610, *p* = 0.009) and Spatial Detachment (*β* = 0.122, t = 2.304, *p* = 0.021) exert significant positive effects on sleep quality, suggesting that individuals who can effectively detach psychologically and physically experience better sleep.

Telepressure has significant negative impacts on Psychological Detachment (*β* = −0.265, t = 5.836, *p* < 0.001) and Spatial Detachment (*β* = −0.153, t = 3.009, *p* = 0.003), yet its direct effect on sleep quality is non-significant (*β* = −0.028, t = 0.572, *p* = 0.567). This indicates that telepressure influences sleep primarily through its detrimental effects on psychological and spatial detachment.

**Figure 2:**
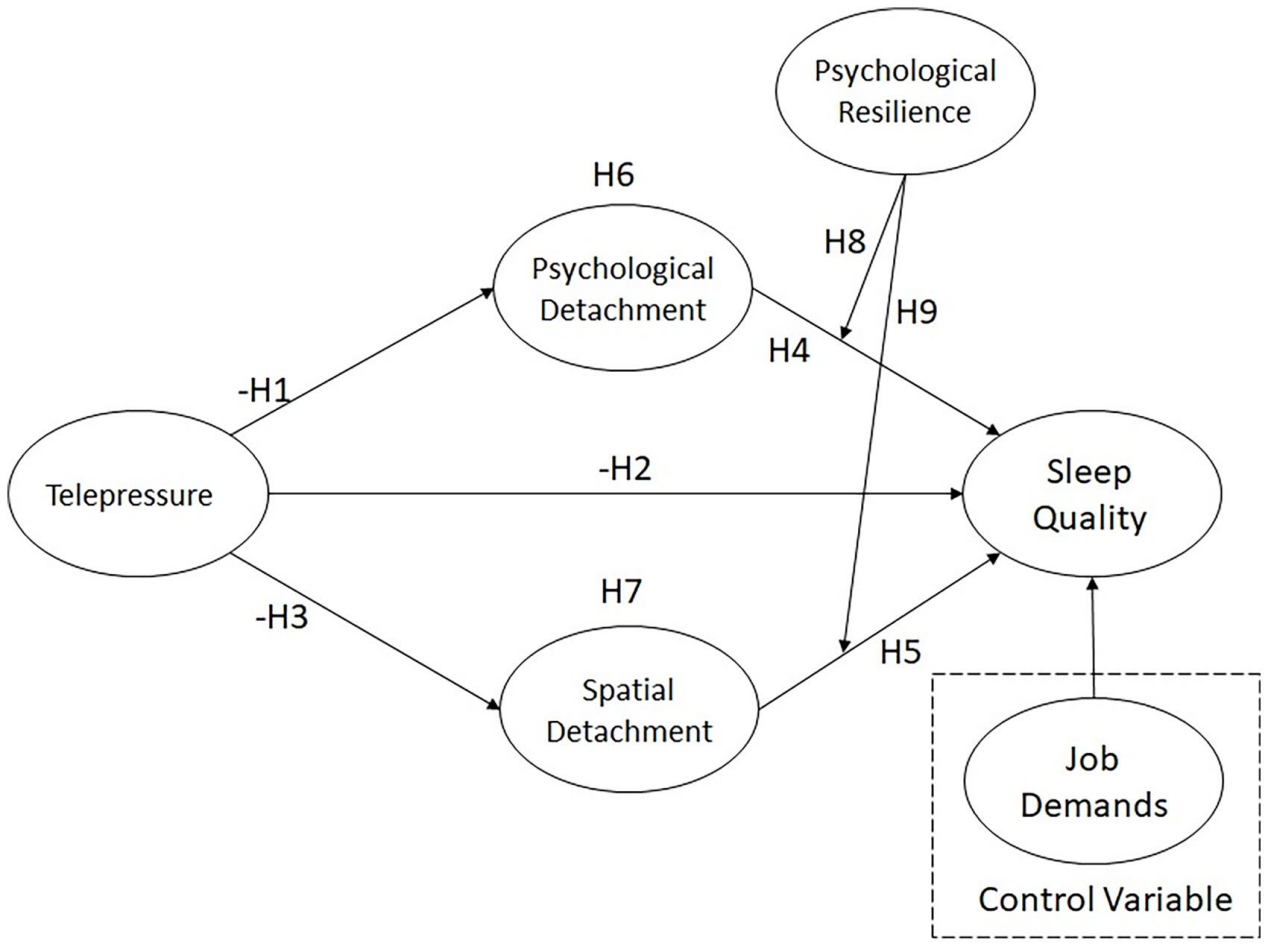
PLS-SEM statistical model.

Overall, high job demands and elevated telepressure reduce individuals’ ability to detach, thereby harming sleep quality; conversely, effective psychological and spatial detachment help maintain good sleep. See Table 5 and Figure 2 for details.

The explanatory power of the structural model was evaluated using the coefficient of determination (*R*^2^). According to the guidelines suggested by Hair et al. (30), *R*^2^ values of 0.75, 0.50, and 0.25 can be described as substantial, moderate, and weak, respectively. In this study, the *R*^2^ value for psychological detachment is 0.070, indicating that telepressure explains 7.0% of the variance in psychological detachment. The *R*^2^ value for spatial detachment is 0.023, suggesting that telepressure explains 2.3% of the variance in spatial detachment. Finally, the R^2^ value for sleep quality is 0.130, indicating that telepressure, psychological detachment, spatial detachment, and the control variable (job demands) jointly explain 13.0% of the variance in sleep quality. Although the explanatory power can be considered relatively modest, such levels are commonly observed in behavioral and psychological research involving complex human behavior, where outcomes are influenced by multiple contextual and individual factors.

The effect size (*f*^2^) was assessed to evaluate the contribution of each predictor to the endogenous constructs. According to Chin (38), *f*^2^ values of 0.02, 0.15, and 0.35 indicate small, medium, and large effects, respectively. As shown in Table 6, telepressure demonstrates a small effect on psychological detachment (*f*^2^ = 0.076) and spatial detachment (*f*^2^ = 0.024). Job demands also show a small effect on sleep quality (*f*^2^ = 0.023). In addition, the interaction between psychological resilience and spatial detachment exhibits a small effect on sleep quality (*f*^2^ = 0.027). The remaining relationships demonstrate negligible effect sizes.

**Table 6:** Effect size (*f*^2^) of the structural model.

Path relationship	*f*2
Job Demands→ Sleep Quality	0.023
Psychological Detachment →Sleep Quality	0.018
Psychological Resilience → Sleep Quality	0.018
Spatial Detachment → Sleep Quality	0.012
Telepressure →Psychological Detachment	0.076
Telepressure → Sleep Quality	0.001
Telepressure →Spatial Detachment	0.024
Psychological Resilience x Psychological Detachment →Sleep Quality	0.003
Psychological Resilience x Spatial Detachment →Sleep Quality	0.027

### 4.6 Mediation analysis

The mediation analysis results show that the indirect effect of Telepressure → Spatial Detachment → Sleep Quality is −0.019 (95% CI = [−0.042, −0.002]), with the confidence interval not containing zero, indicating statistical significance. Similarly, the indirect effect of Telepressure → Psychological Detachment → Sleep Quality is −0.039 (95% CI = [−0.073, −0.009]), which is also significant. These findings indicate that telepressure indirectly impairs sleep quality by reducing individuals’ psychological and spatial detachment abilities.

Although the direct effect of telepressure on sleep quality is not significant, the two significant negative indirect effects confirm that both psychological and spatial detachment serve as critical mediators in this relationship. See Table 7 for details. However, the direct effect of telepressure on sleep quality was not significant (*β* = −0.028, *p* = 0.567). According to the mediation criteria suggested in PLS-SEM literature, this pattern indicates full mediation. Specifically, telepressure influences sleep quality indirectly through both psychological detachment and spatial detachment.

**Table 7:** Mediation analysis.

Path relationship	Original sample	2.50%	97.50%
Telepressure → Spatial Detachment →Sleep Quality	−0.019	−0.042	−0.002
Telepressure → Psychological Detachment →Sleep Quality	−0.039	−0.073	−0.009

### 4.7 Moderation analysis

**Figure 3:**
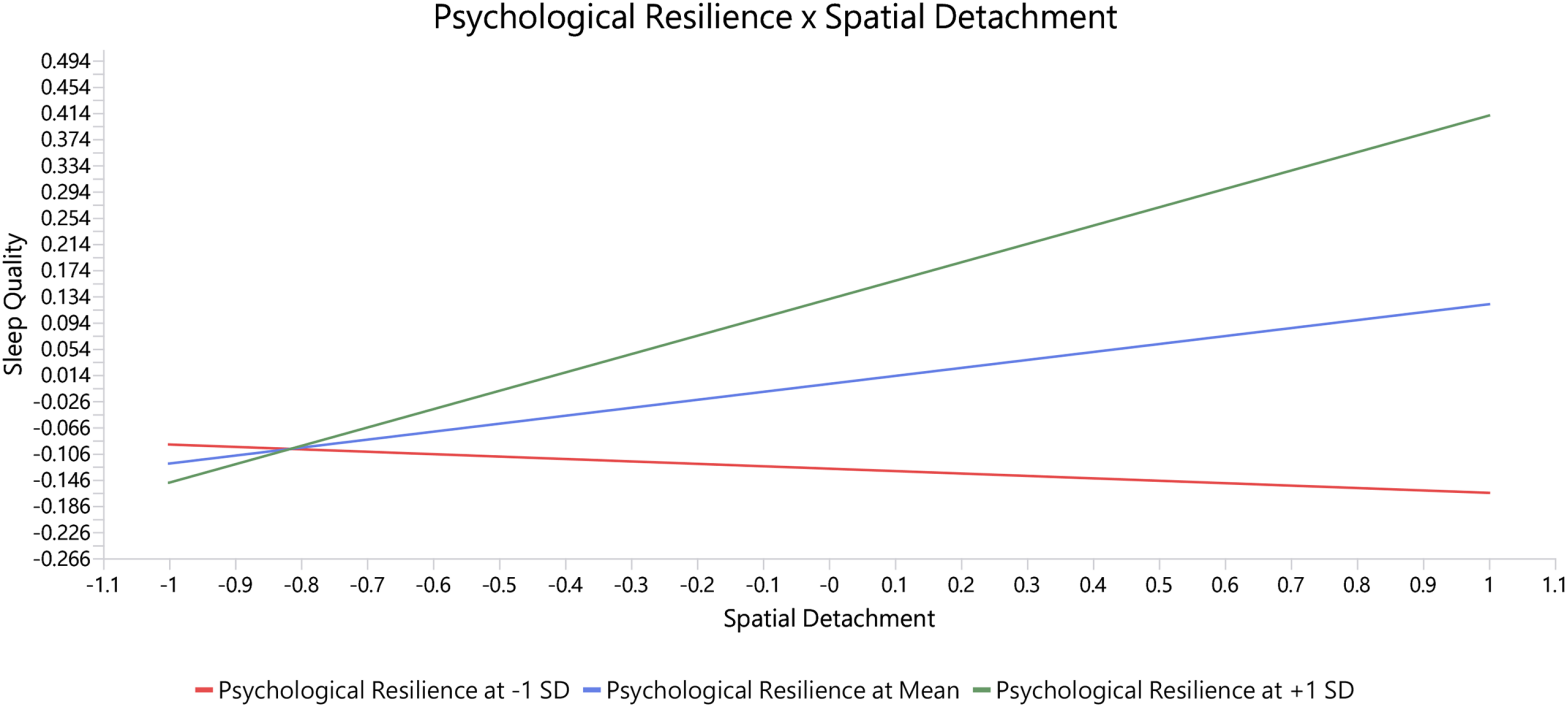
Illustration of the moderating effect of psychological resilience × spatial detachment on sleep quality.

The moderation analysis results show that the interaction term Psychological Resilience × Psychological Detachment → Sleep Quality is not significant (*β* = −0.053, t = 0.919, *p* = 0.358), indicating that psychological resilience does not alter the effect of psychological detachment on sleep quality. However, the interaction term Psychological Resilience × Spatial Detachment → Sleep Quality is significant (*β* = 0.159, t = 2.734, *p* = 0.006), suggesting that psychological resilience positively moderates the relationship between spatial detachment and sleep quality. Specifically, individuals with higher psychological resilience experience greater improvements in sleep quality when they are able to effectively detach physically from work. See Table 8 and Figure 3 for details.

**Table 8:** Moderation analysis.

Path relationship	Path coefficient	Standard deviation	*t*-value	*p-*value
Psychological Resilience x Psychological Detachment → Sleep Quality	−0.053	0.058	0.919	0.358
Psychological Resilience x Spatial Detachment →Sleep Quality	0.159	0.058	2.734	0.006

## 5 Discussion and conclusion

### 5.1 Summary of key findings

#### 5.1.1 The impact of being “always online” on psychological and spatial detachment

The findings of this study indicate that telepressure (the pressure to be constantly available) exerts significant negative effects on both psychological detachment and spatial detachment, supporting the core propositions of the Stressor–Detachment Model and Boundary Theory. According to Sonnentag and Fritz (5), persistent work demands hinder individuals from achieving psychological “disengagement” during non-work hours, thereby impairing recovery processes and overall well-being. Consistent with this perspective, the present study shows that when individuals face expectations of immediate responsiveness and social norms of constant availability, they struggle to mentally disconnect from work even after leaving the workplace. This difficulty in detaching results in sustained cognitive activation and challenges in achieving effective recovery.

This result is consistent with the findings of Barber and Santuzzi (4) and Barber et al. (3), which examined general organizational employee samples and similarly showed that telepressure suppresses after-work psychological detachment and increases risks of fatigue and insomnia. However, by focusing on digital nomads, the present study expands the theoretical applicability into a new labor context. Unlike employees working in fixed office settings, digital nomads operate across multiple time zones and geographical locations, facing highly blurred work–life boundaries and more persistent pressures to respond to messages. As a result, psychological detachment is disrupted not only at a cognitive level but also through the overlap of time and space, indicating that telepressure exerts even more profound effects on recovery mechanisms in emerging forms of mobile and boundaryless work.

In addition, this study provides empirical evidence for the significant negative effect of telepressure on spatial detachment, addressing a gap in the literature that has largely overlooked physical and behavioral boundary strategies. Prior studies have focused mainly on psychological processes (3, 39), with limited attention to how individuals use spatial or behavioral strategies to maintain recovery boundaries. According to Matthews et al. (15) and Kossek et al. (24), spatial detachment can be achieved through establishing fixed workspaces, turning off devices, or creating offline rituals to reduce role interference. However, the present findings reveal that under environments of high telepressure, such behavioral strategies frequently fail because work-related messages infiltrate all physical spaces—including bedrooms, vehicles, and public areas—resulting in the “re-workification” of non-work environments and the erosion of essential recovery conditions.

#### 5.1.2 The mediating roles of psychological detachment and spatial detachment

This study confirms the significant mediating effects of psychological detachment and spatial detachment in the relationship between telepressure and sleep quality, supporting the core assumptions of the Stressor–Detachment Model (5) and recovery theory. The results indicate that although the direct effect of telepressure on sleep quality is not significant, it indirectly undermines sleep quality by reducing individuals’ capacities for psychological and spatial detachment. This finding suggests that the negative impacts of telepressure do not manifest immediately; instead, they accumulate through disruptions to recovery processes.

On the psychological level, the expectation of constant availability triggered by telepressure makes it difficult for individuals to stop thinking about work or emotionally disengage during non-work hours. This persistent cognitive occupation results in insufficient psychological detachment, reducing opportunities for relaxation and recovery, ultimately harming sleep quality. This finding aligns with Sonnentag and Fritz (20), Sonnentag and Fritz (5), and Barber and Santuzzi (4), all of whom highlight psychological detachment as a crucial link in the stress–health process. Notably, this study further demonstrates that psychological detachment plays an even more salient role among digital nomads. Given their highly autonomous yet unstable work patterns, the boundaries between work and non-work time are especially blurred, making psychological detachment more difficult and the cumulative psychological consequences of telepressure more pronounced.

On the spatial level, this study finds that telepressure indirectly affects sleep quality by weakening spatial detachment, indicating that the breakdown of spatial boundaries is another key mechanism contributing to sleep disturbances. When work messages are continuously accessible, individuals tend to engage in work tasks in all types of spaces—such as bedrooms or public areas—leading to the re-workification of environments that are originally intended for rest. This is consistent with Matthews et al. (15) and Kossek et al. (24), who argue that inadequate spatial or behavioral boundary strategies undermine recovery and well-being. By empirically demonstrating this mechanism, the present study fills a gap in prior literature, offering empirical evidence of the dual mediating roles of psychological and spatial detachment in the telepressure–sleep quality relationship.

#### 5.1.3 The moderating role of psychological resilience

The results of this study show that psychological resilience significantly and positively moderates the relationship between spatial detachment and sleep quality, but does not significantly moderate the relationship between psychological detachment and sleep quality. This partially supports the proposition from Conservation of Resources (COR) Theory (18) and the Job Demands–Resources (JD–R) Theory (16), which states that personal resources can buffer the adverse effects of job demands.

Psychological resilience is considered a developable personal resource (26) that helps individuals maintain functioning and emotional stability in stressful environments. The findings indicate that individuals with higher psychological resilience are better at maintaining physical boundaries and behavioral routines. Even when facing strong expectations of availability caused by telepressure, they are more capable of adhering to offline rituals and spatial separation, thereby achieving better sleep quality. This aligns with Sonnentag and Fritz (5) and Xanthopoulou, Bakker, Demerouti, and Schaufeli (40), both of which emphasize that personal resources enhance recovery processes and promote well-being.

In contrast, the moderating effect of psychological resilience on the relationship between psychological detachment and sleep quality is non-significant. This may be because psychological detachment is an internal cognitive process that is more easily disrupted by continuous cognitive activation and ruminative thoughts triggered by telepressure. In other words, when work-related messages repeatedly intrude on one’s mind, even individuals with high resilience may struggle to fully block cognitive intrusions. This finding echoes Barber et al. (3), who noted that in “always-on” work environments, successful psychological detachment often requires external structural support rather than relying solely on individual psychological traits.

### 5.2 Theoretical contributions

This study centers on the telepressure–detachment–sleep quality pathway and develops an integrated model of work stress and recovery processes in the digital era. The findings make several contributions to the literature on digital work stress, recovery processes, and occupational health.

First, this study extends the applicability of the Stressor–Detachment Model and Recovery Theory to contemporary digital work environments. Prior research has predominantly focused on traditional office settings and has overlooked how the “always-on” culture emerging from mobile work and remote collaboration erodes the recovery process (5). By incorporating telepressure into the model, this study demonstrates that telepressure is not only a psychological stressor but also a technologically mediated digital demand. It indirectly harms sleep quality by simultaneously undermining both psychological and spatial detachment. This finding fills a theoretical gap by clarifying how technology-driven stressors translate into health outcomes.

Second, the study is one of the few studies to incorporate both psychological detachment and spatial detachment into a dual-pathway recovery model. Previous scholarly discussions largely regarded detachment as a single cognitive process (e.g., mental disengagement), neglecting its physical and behavioral dimensions (15). Drawing on Boundary Theory, this research differentiates between the mechanisms of psychological and spatial detachment and empirically tests their mediating roles between telepressure and sleep quality. In doing so, the study expands both the depth and structure of stress–recovery models, rendering the detachment construct more comprehensive and operationalizable.

Third, by introducing psychological resilience as a moderator, the study advances the integrated application of the Job Demands–Resources (JD–R) Theory and Conservation of Resources (COR) Theory. The findings show that psychological resilience strengthens the positive effect of spatial detachment on sleep quality, confirming its buffering function within the “demands–recovery–health” chain (18, 28). This result not only reinforces the idea that personal resources enhance recovery effectiveness but also highlights the complementary and amplifying effects among different types of resources (psychological vs. behavioral), enriching the dynamic perspective of the JD–R framework.

Finally, this study contributes theoretically by employing digital nomads as the empirical context for extending stress and recovery theories. Prior research in this domain has focused primarily on organizational employees. By examining a highly mobile group with weak geographic anchoring, this study reveals that digital nomads are more vulnerable to the erosion of both psychological and spatial detachment under telepressure. This finding identifies unique health risks faced by emerging nontraditional labor groups and enhances the external validity of stress-management theories within boundaryless, digital, and mobile work settings.

### 5.3 Practical implications

The findings of this study provide several practical insights for promoting workplace health and sustainable management in digital work environments. The results suggest that telepressure may create hidden health risks in technology-mediated work contexts, particularly by weakening employees’ psychological and spatial detachment and thereby impairing sleep quality and recovery. From an organizational perspective, these findings highlight the importance of developing digital communication norms that support boundary management. For example, organizations may benefit from introducing communication policies such as the right to disconnect, limiting non-urgent communication during non-work hours, or using automated response systems to reduce expectations of immediate replies. Such practices may help employees maintain clearer boundaries between work and personal time in digitally connected environments. In addition, managerial behavior plays a critical role, as leaders’ communication patterns can signal expectations regarding availability and responsiveness in remote work settings.

Second, the results indicate that organizational environments and work design may influence employees’ ability to experience effective detachment and recovery. Beyond psychological rest, environmental cues and organizational practices can function as mechanisms that facilitate recovery experiences. For instance, organizations may consider establishing designated quiet periods for non-work communication, creating spaces that symbolically separate work and rest, or providing digital tools that support employees in monitoring rest intervals. These practices may help reduce work intrusions into private life and support employees’ post-work recovery processes, thereby contributing to healthier and more sustainable work patterns.

Third, the findings also highlight the importance of individual resources, particularly psychological resilience, in managing telepressure. Individuals with higher levels of psychological resilience appear better able to maintain spatial separation and recovery routines under conditions of high telepressure, which is associated with improved sleep outcomes. From a practical perspective, organizations may support employee well-being by offering resilience-building initiatives, such as stress management training, mindfulness-based programs, or workshops aimed at strengthening psychological resources. At the same time, employees may benefit from developing personal boundary-management strategies, such as disabling notifications after work hours or establishing consistent end-of-work routines, which can help reduce technological intrusions and facilitate more effective detachment from work-related demands.

Although the hypothesized relationships were statistically significant, the explanatory power of the model should be interpreted with caution. The R^2^ values for psychological detachment (0.070), spatial detachment (0.023), and sleep quality (0.130) indicate that the predictors included in the model explain a modest proportion of variance in these outcomes. This suggests that while telepressure represents an important antecedent within the digital work stress–recovery process, detachment and sleep quality are likely influenced by a broader set of contextual, behavioral, and individual factors beyond those captured in the present model. For instance, factors such as work schedule irregularity, chronotype differences, living environment, housing conditions, and social support may also play meaningful roles in shaping recovery experiences and sleep outcomes among digital nomads. Therefore, the current findings should be interpreted as identifying one theoretically relevant pathway rather than providing a comprehensive explanation of sleep quality in digitally mobile work contexts.

### 5.4 Limitations and future research

Although this study provides meaningful theoretical and empirical insights, several limitations should be acknowledged. First, this study adopted a cross-sectional research design, which restricts causal inference and does not capture temporal changes among telepressure, detachment, and sleep quality. Although the proposed relationships were grounded in established theoretical frameworks, the data do not allow for examination of dynamic processes over time. Future research could employ longitudinal designs or experience sampling methods to better observe the temporal dynamics of telepressure, recovery experiences, and sleep outcomes. Second, sleep quality in this study was measured using the self-reported Pittsburgh Sleep Quality Index (PSQI). Although the PSQI is a widely validated and commonly used instrument in sleep and health research, it primarily reflects individuals’ subjective perceptions of sleep quality. Future studies could incorporate objective physiological measurements, such as polysomnography or wearable sleep-monitoring devices, to provide more precise assessments of sleep patterns and further validate the relationships identified in this study. Third, the relatively modest R^2^ values for psychological detachment, spatial detachment, and sleep quality suggest that the predictors included in the model explain only a limited proportion of variance in these outcomes. This indicates that recovery experiences and sleep quality are likely influenced by a broader set of contextual, behavioral, and individual factors beyond those examined in this study. For example, chronotype differences, work schedule irregularity, housing conditions, social support, and lifestyle habits may also play meaningful roles in shaping detachment and sleep outcomes among digital workers. Future research could incorporate these additional variables to further refine the explanatory framework. Fourth, although the sample consisted of individuals engaged in location-independent work across multiple cities, a substantial proportion of respondents reported relatively low weekly remote work hours. This suggests that the sample may include hybrid or lower-intensity forms of digitally mobile work rather than exclusively full-time digital nomads in a strict sense. Therefore, the findings may be more appropriately generalized to a broader population of digitally mobile and flexible remote workers. Future research is encouraged to adopt stricter classification criteria, such as minimum remote work intensity or frequency of geographic mobility, to improve conceptual precision. Finally, the sample consisted mainly of digital nomads whose high mobility and flexible work arrangements may limit the generalizability of the findings. Future research should include broader remote-working populations and cross-cultural samples to examine how institutional environments and social support systems shape the telepressure–recovery relationship. Overall, future studies may further advance the demands–recovery–health framework through longitudinal tracking, multilevel analyses, and cross-context comparisons.

## 6 Conclusion

This study should also be interpreted in light of its sociocultural context. Specifically, the findings are based on digital nomads in China, where work norms are often characterized by stronger expectations of responsiveness, collectivist values, and hierarchical organizational cultures. Compared to Western contexts, where work–life boundaries and individual autonomy may be more strongly emphasized, Chinese workers may experience higher levels of telepressure and weaker boundary control. These sociocultural differences may influence the strength and mechanisms of the relationships examined in this study. Therefore, caution should be exercised when generalizing the findings to Western contexts. Future research is encouraged to conduct cross-cultural comparative studies to examine whether the observed mechanisms hold across different institutional and cultural environments.

## Data Availability

The datasets presented in this study can be found in online repositories. The names of the repository/repositories and accession number(s) can be found in the article/supplementary material.
